# Feasibility testing of a home-based exercise intervention in children with cerebral palsy who are ambulant—a study protocol of the HOME-EX study

**DOI:** 10.3389/fdgth.2026.1811789

**Published:** 2026-05-12

**Authors:** Trille Jakobsson, Katarina Lauruschkus, Björn A. Johnsson, Åsa Andersson, Ola Hansson, Robert Holmberg, Åsa B. Tornberg

**Affiliations:** 1 Department of Health Sciences, Lund University, Lund, Sweden; 2 Department of Nursing and Integrated Health, Kristianstad University, Kristianstad, Sweden; 3 Itacih, Lund, Sweden; 4 Department of Environmental- and Biosciences, FIH, Halmstad University, Halmstad, Sweden; 5 Department of Clinical Sciences, Malmö, Lund University, Malmö, Sweden; 6 Department of Psychology, Lund University, Lund, Sweden

**Keywords:** cerebral palsy, children, eHealth, exercise intervention, mixed methods design, participatory approach

## Abstract

Children gain increased health and well-being by participating in physical activity. Children with cerebral palsy who are ambulatory (CP-A) are known to be less physically active than children without physical disabilities, making them an important group of children to support in becoming more physically active. Assistive technologies, such as eHealth, may support physical exercise in children with CP-A. The overall aim of this study protocol will be to describe the procedures of the HOME-EX feasibility study to develop, feasibility test, and map the conditions for future implementation of a home-based exercise intervention using an eHealth solution in children with CP-A.

**Methods:**

The HOME-EX feasibility study will use a mixed methods approach with an adapted participatory design. The study is informed by “The behaviour change wheel” framework to support health related behavioural changes. The development and the feasibility testing will follow three phases: 1) development; 2) refinement; and 3) finalising, where the children will perform two six-week periods of home-based exercises using an eHealth solution with eight weeks of washout in-between. Ten children with CP-A and ten children without disabilities, aged 10–16 years, living in the southern regions of Sweden will be recruited. The feasibility of the eHealth solution, the home-based exercise intervention, and the exercise testing will be assessed throughout the study. Field notes, questioners, data from the eHealth solution and semi-structured interviews will be used to collect data.

**Discussion:**

Findings from the HOME-EX study have the potential to increase knowledge about the development and feasibility of a home-based exercise intervention, supported by the eHealth solution, to promote exercise in children with CP-A.

**Clinical Trails Registration:**

https://clinicaltrials.gov/expert-search?term=NCT07025694, identifier NCT07025694.

## 1 Introduction

Cerebral palsy (CP) is the most common physical disability in childhood; approximately 1.6/1000 children have CP with affected muscle tone, movement, and motor skills, often accompanied by pain, epilepsy, and intellectual, communicational, and behavioural impairment (1). The degree of motor impairment in CP is highly variable and is classified according to the five-level Gross Motor Function Classification System Expanded & Revised scale (GMFCS-E&R) (2). Children with CP who are non-ambulant (GMFCS-E&R levels IV and V) cannot stand or walk independently, whereas children with CP who are ambulant (CP-A; GMFCS-E&R levels I–II) can walk and stand independently. Children with CP GMFCS-E&R level III can either walk and/or stand independently or with a walking aid.

Children gain increased health and well-being by participating in physical activity, including children with CP-A. According to the World Health Organisation's (WHO) recommendations, children should be physically active at least 60 min per day, regardless of whether or not they are living with a disability (3) Physical activity is defined as any bodily movement that increases energy expenditure (4), while exercise training is a planned and structured physical activity (4). A healthy lifestyle, including being physically active, is normally established during childhood and adolescence, making children an important group to study and target (5). Only 20% of all children and adolescents in Sweden meet these recommendations (6). Furthermore, children with CP are known to be even less physically active (7), making this an important group of children to support in becoming more physically active.

Multiple exercise training sessions improve cardiopulmonary functions, adapt muscle fibre distribution, improve metabolic parameters linked to cardiovascular risk, and reduce the level of inflammatory markers (8). Furthermore, there is evidence that exercise training has a beneficial effect on neuronal and cognitive functions in children (9). Levels of the neural growth factors−brain derived neurotropic factor (BDNF), nerve growth factor (NGF), and vascular endothelial growth factor (VEGF)−were found to be increased by exercise, which may be a mechanism behind the positive effect on the brain and cognitive function in children without physical disabilities (TD) (10) training influence levels of neural growth factors, highlighting an important knowledge gap.

To map the effects of exercise training in children, robust study designs are needed. In recent years, there has been increasing interest in the potential of assistive technologies (11) such as eHealth to promote exercise in various populations (12), including individuals with disabilities (13). eHealth refers to the use of digital technologies, such as mobile applications, wearable devices, and online platforms, to deliver health-related services and information (14). The potential of eHealth in children with CP-A is promising in terms of supporting exercise by improving accessibility to healthcare professionals and reducing time-consuming transportation (15–18). Previous studies, including children with CP-A, have shown associations between eHealth and improvements in several motor and functional outcomes, including gross motor function, functional strength, walking endurance, occupational performance, dexterity, and bimanual hand function (15, 17, 19). However, existing eHealth research in children with CP-A has largely focused on upper limb function and functional motor outcomes (20). Consequently, to the best of our knowledge, the potential of eHealth to support exercise training to improve cardiopulmonary functions and reduce non-communicable diseases in children with CP-A remains unexplored.

To further explore how eHealth could potentially support home-based exercise training in children with CP-A, an eHealth solution—developed from an already existing and operational eHealth solution (21, 22)—will be used in this study. The eHealth solution includes: a) an eHealth application (exer-app) on a tablet computer used by the children and their family caregivers, which securely connects to b) a server and computer within the healthcare system used by the researchers (21, 22).

The HOME-EX study takes a bio-psychosocial perspective of human beings and human behaviour, anticipating that human behaviour is a complex phenomenon consisting of physiological, psychological, and environmental components (23). Health will be seen as an interaction between the child and the environment surrounding the child, such as family, health policies, and societal attitudes (24). The research project design relies on the framework “The behaviour change wheel” to enable health related behavioural change, which consists of three levels: 1) sources of behaviour (children with CP-A); 2) intervention function (home-based exercise); and 3) policy categories (WHO guidelines) (25). The eHealth solution will contribute to a better alignment between the sources of behaviour and the intervention function. Furthermore, information from the eHealth app may contribute to the evaluation of the exercise intervention, which in turn could contribute to better decision-making at the interface between intervention functions and policy.

Applying an ecological systems theory is appropriate when exploring how to support exercise through an eHealth solution in children with CP-A to understand the complex interplay between the child and his/her multiple levels of surrounding environment (26). The HOME-EX study will address the children, their family caregivers, and professional fitness personal (i.e., personal trainers) to investigate whether an eHealth solution could beneficially enhance home-based exercise in children with CP-A.

The overall aim of this study protocol is to describe the procedures of the HOME-EX feasibility study to further develop, feasibility test, and map the conditions for future implementation of a home-based exercise intervention using an eHealth solution in children with CP-A.

## 2 Methods

### 2.1 Design

**Figure 1:**
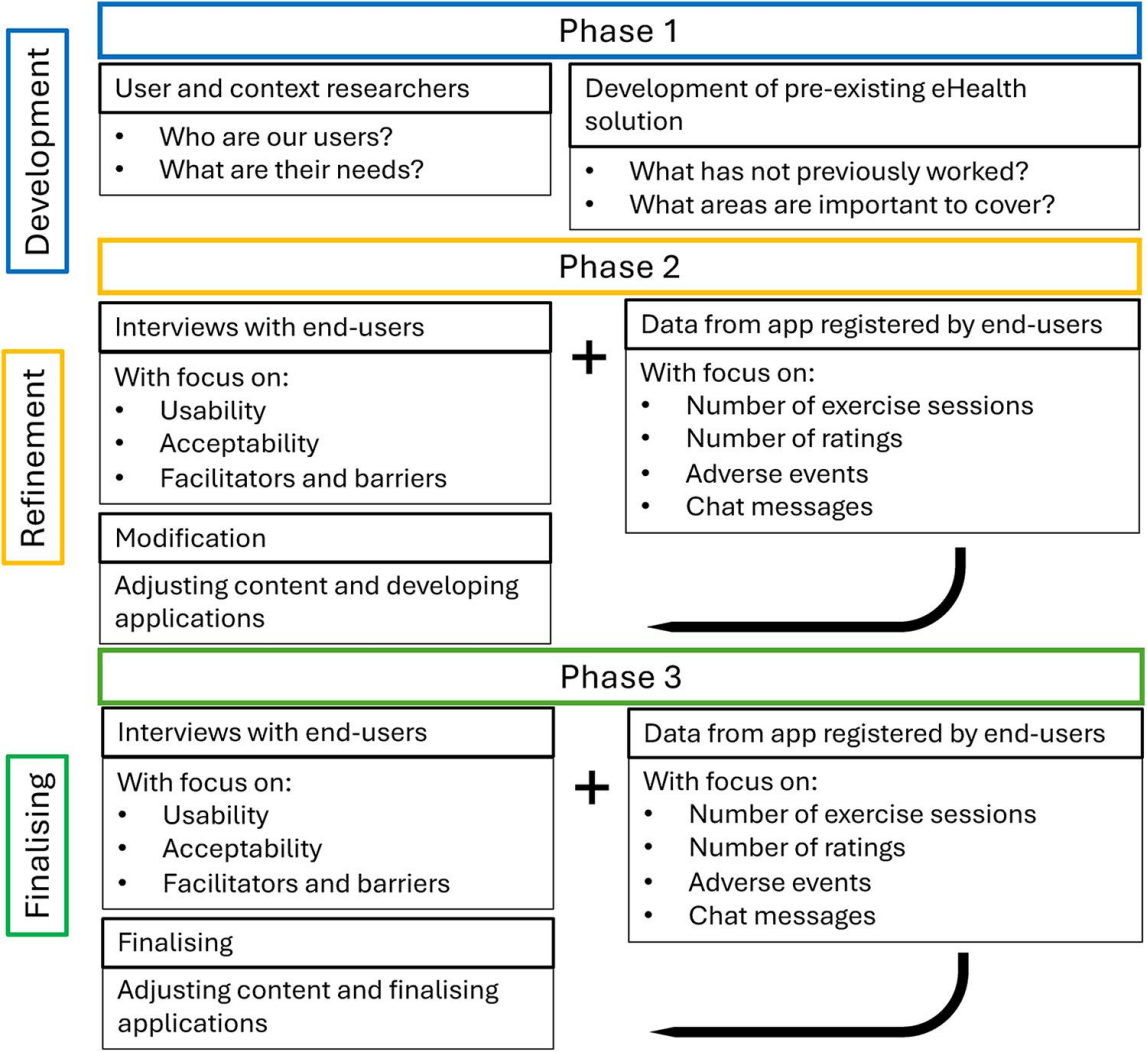
The three phases of adapted participatory design for development and feasibility testing of the eHealth solution, mapped to the stages of design thinking.

The present study protocol is reported according to the SPIRIT checklist (27) (supplementary S1), supplemented by the Consolidated criteria for reporting qualitative research (COREQ) checklist (28) (supplementary S2) and the Good Reporting of A Mixed Methods Study (GRAMMS) checklist (29) (supplementary S3). A convergent mixed methods approach (30) with a participatory design (31) will be used, collecting both quantitative and qualitative data (30). Data will be analysed separately and then integrated into the discussion for joint interpretation and insights on feasibility (30). Participatory design ensures end-users' involvement in adapting the eHealth solution, exercise intervention, and exercise testing. The development and feasibility testing will follow three phases: 1) development, 2) refinement, and 3) finalising (Figure 1) (31). The exercise intervention will consist of two six-week periods of home-based exercises using the eHealth solution with eight-weeks of washout in-between. Furthermore, the exercise testing will be performed before and after each exercise period.

### 2.2 Methodological frameworks

The research project builds on the methodological framework on complex interventions from the Medical Research Council (MRC) (32), which includes four phases: development, feasibility/piloting, evaluation, and implementation, with the core elements of considering context, refining programme theory, engaging stakeholders, identifying key uncertainties, refining intervention, and considering the economics of each phase. To evaluate the feasibility of study design information about process, scientific-, management-, and resource-based uncertainties (33) will be collected. Additionally, the Non-Adoption, Abandonment, and Challenges to Scale-up, Spread, and Sustainability of Health and Care Technologies (NASSS-CAT) tools (34) will be used to map conditions for future implementation throughout the entire research project. To systematically assess the likelihood of adoption and scaling up of the eHealth solution, the NASSS-CAT will be used as a tool for risk assessment at different stages of the project.

### 2.3 Participants

A purposely recruited sample of participants will be conducted until saturation is reached (35). Saturation will be seen as to be reach when no new information is retrieved from the semi-structured interviews. 20 children, ten children with CP-A and ten children who are TD will be anticipated to be included, aged 10–16 years. The children will be aged matched. The sample size of 20 participants is consistent with common practice in pilot and feasibility studies, where the primary aim is to assess feasibility rather than effectiveness (36). Children who are TD will be included in the feasibility study to assess the feasibility of recruitment and all the study procedures in both groups, as a potential future lager study assessing intervention effects will require both children with CP-A and children who are TD as controls.

All participants will be recruited from the southern regions of Sweden. Children younger than 15 will be recruited via their parents to participate in the study. Children 15–16 years old can consent or refuse to consent to participate in the study without their parents' consent. Social media such as Facebook, Instagram, and LinkedIn will be used for recruitment. Additionally, the National Association for Disabled Children in Sweden and the Habilitation Services will be used for children with CP.

*Inclusion criteria:* children with CP-A defined as GMFCS-E&R I–II and children who are TD and not participating in any regular and planned leisure time physical exercise will be included in the study.

*Exclusion criteria:* Ambulant children with CP, walking with a walking aid (GMFCS-E&R III); non-ambulant children with CP (GMFCS-E&R IV and V); ambulant children who are TD with other neurological diagnoses, metabolic diagnoses, orthopaedic disabilities, asthma, heart disease, cognitive impairment, or indigestion of regular medication.

### 2.4 Settings

The exercise intervention will be performed in the children's homes. Each child will have a cycle ergometer (Monark Exercise AB, Sweden), heart rate monitor (Polar, Finland), and an android tablet computer delivered to their home. All equipment will be personally adjusted. The exercise testing will be performed in an exercise lab at Lund University, Lund.

### 2.5 Home-based exercise intervention

**Figure 2:**
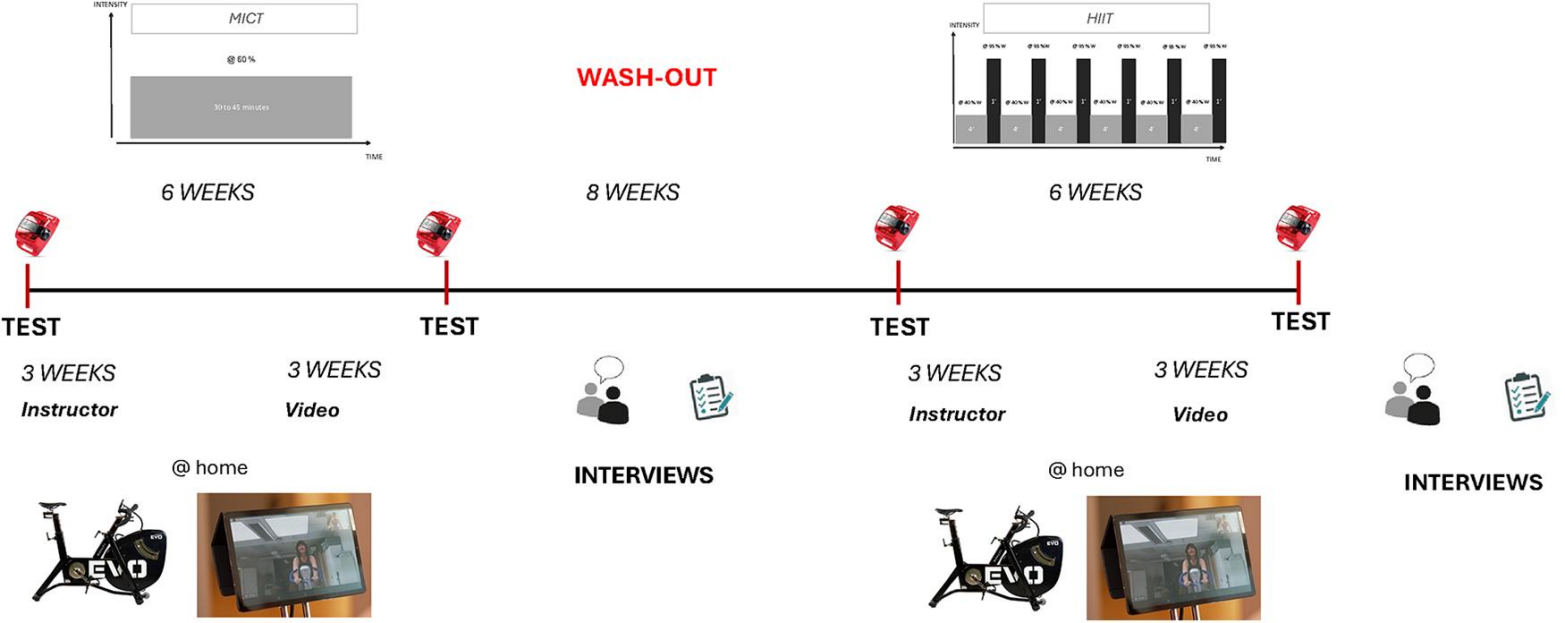
The feasibility study, with a cross-over design including test-points, exercise training periods, wash-out, and timepoints of the interviews.

Home-based supervised exercise training will consist of two different exercise modalities, Moderate Intensity Continuous Training (MICT) and High Intensity Interval Training (HIIT) (37), with individually adapted intensities based on the results from the exercise testing. Each exercise period will consist of six weeks with an eight-week washout in-between, starting with MICT. The exercise periods start and end with exercise testing (Figure 2).

The MICT exercise training will consist of moderate intensity/longer (60%Wpeak) duration exercise (37) carried out three times per week—Tuesday, Thursday, and Saturday during the three-week period of supervision via video conferencing, and at timepoints chosen by the child with at least one day in between sessions during the periods of video-supervision. Exercise will consist of continuous biking at 60%Wpeak for a total time of 30 min (Figure 2).

The HIIT exercise training will begin by using a protocol consisting of bouts of high-intensity (95%Wpeak) exercise lasting for 60 s (37). These bouts will be interspersed by four minutes of low-intensity (40%Wpeak) exercising (37). There will be five bouts per session, with the same weekly plan as for MICT (Figure 2).

### 2.6 eHealth solution

**Figure 3:**
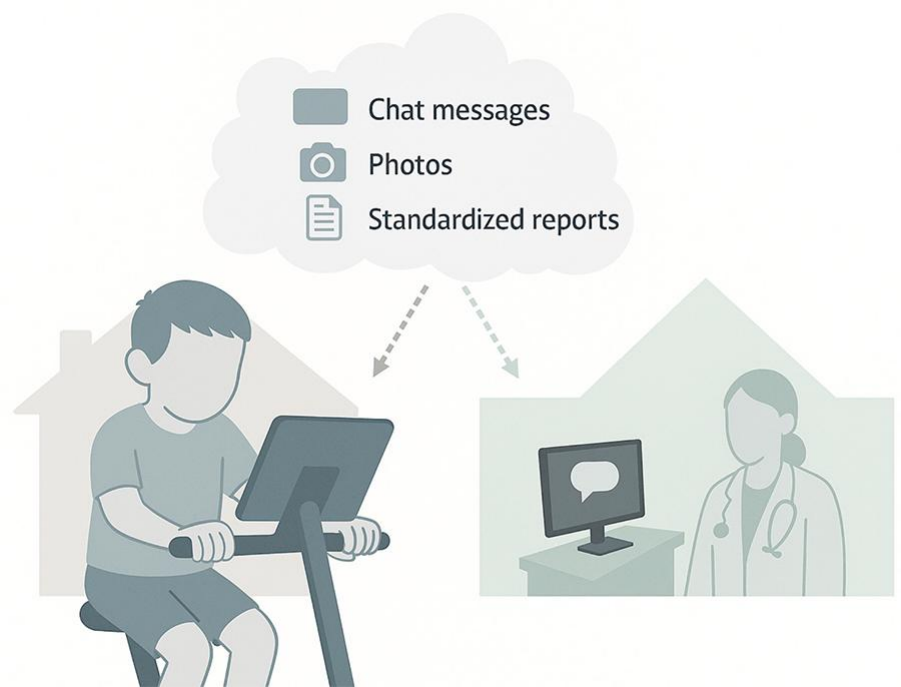
The composition of the eHealth solution used to deliver the exercise training. The eHealth app on the android tablet computer will be used by participating children and their families, and the interface used by researchers. All data will be stored at a centralised server.

The supervised exercise training will be delivered through the eHealth solution. The children and families will use the eHealth app on the android tablet computer, that builds on the technical middleware framework PalCom (38). The app connects to a centralised server where data from the eHealth app are stored encrypted. The server environment is based on the results of the previous research project IT Support for Advanced Care in the Home (39). Researchers will connect to the server by using the web browser of their ordinary workstation computer, thereby enabling them access to the data and to correspond. The eHealth app is equipped with a SIM-card enabling constant internet connection. The eHealth solution was first established in 2016 in Region Skane (22) and refined in several developmental circles (31) up until 2023 (Figure 3).

Planned functions in the eHealth solution include text messaging (chat), video calls, calendar, recordings of how the exercise is perceived, delivery of the exercise sessions, and recordings of heart rates and workloads during the exercise session. It will be possible for the child and family to reach out to the researcher via the text messaging function, and vice versa, to increase compliance with the intervention. In the calendar, the children will be instructed to note (by ticking a box) if they have performed the exercise sessions, making it possible to follow compliance guidelines. The children will also be instructed to score their experience of the exercise after each session. The exercise intervention is planned to be delivered in two different ways: a) supervised by an experienced personal trainer at fixed timepoints via video conferencing meetings, and b) via pre-recorded videos with the same personal trainer to be used at timepoints convenient for the child and family. Heart rate assessment (Polar, Finland) and workload will be recorded via cycle ergometers (Monark Exercise AB, Sweden).

### 2.7 Feasibility of the effectiveness testing of the exercise intervention

Before and after each exercise period, effectiveness testing will be performed. Prior performing the effectiveness testing, the children will be offered the chance to watch a video describing the procedure and the different tests.

The effectiveness testing comprises the following procedures and tests: when the children arrive in the lab, their weight and height will be measured, followed by answering questions about physical activity, pain, bowel movements (40), and quality of life (41).

Body composition will be assessed by bioimpedance (InBody S10, South Korea). Passive range of motion (pROM) (42) and spasticity (43) in the legs will be assessed according to the CPUP protocol (supplementary S4).

Venous and capillary blood samples will be collected before and after the exercise tests. As part of the feasibility assessment, procedures for blood sampling will be evaluated in preparation for potential future study investigating exercise-induced changes in BDNF and other growth factors. Before coming to the lab, the children will use an EMLA plaster (Aspen Pharma Trading Limited, Dublin, Ireland) containing 25 mg lidocaine and 25 mg prilocaine as pain relief, on the back of their hands and on their arm folds. The EMLA plaster should be applied at the four sites at least one hour, and no more than five hours, before the lab visit. Changes in biomarkers will be assessed in the following categories: 1) neural growth factors, 2) the IGF-axis, 3) the HPA-axis, 4) inflammatory markers, and 5) insulin sensitivity for blood glucose and for blood free fatty acid metabolism; e.g., insulin, glucose, lactate and free fatty acids (supplementary S5).

**Figure 4:**
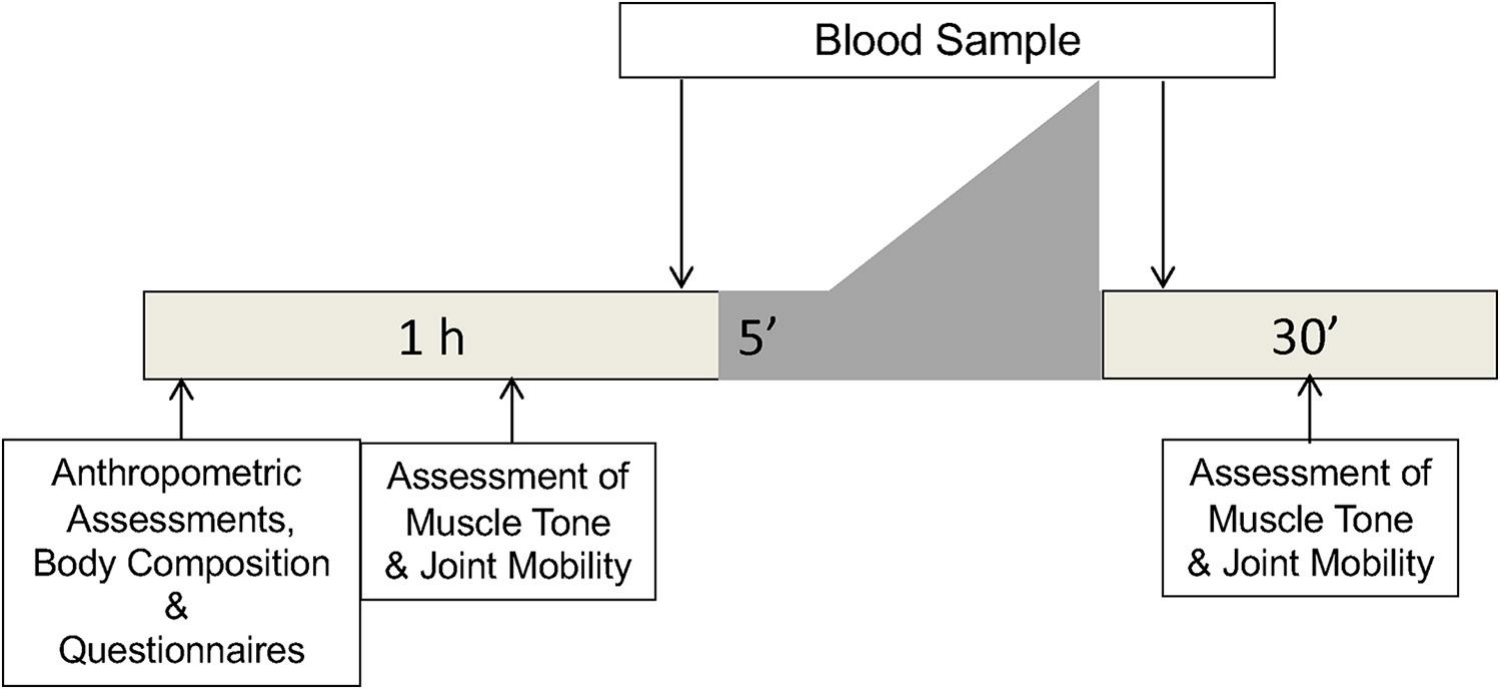
The effectiveness testing of children with CP-A and TD children before and after each exercise period. The dark grey area shows the combined steady state and progressive incremental exercise test, initiated by cycling on a cycle ergometer.

Subsequently, the children will perform a combined steady state and progressive incremental exercise test until exhaustion is described in Figure 2. This exercise test will be initiated by cycling on a cycle ergometer for 6 min at 30 W, followed by an increase of 10 W/min until exhaustion (44) (Figure 4). Indirect calorimetry will be used during a single session of high intensity exercise; i.e., during a combined steady state and progressive incremental exercise test until exhaustion (Figure 4), to assess markers of ventilation, circulation, fat versus carbohydrate oxidation, and energy expenditure during exercise (supplementary S6). The results from the exercise test will be used to set the individually adapted exercise intensities during MICT and HIIT.

After the second blood sample, pROM and spasticity assessment, the children will be asked to wear an accelerometer (GT3X+, ActiGraph, Pensacola, USA) for seven consecutive days. The accelerometer will be worn before and after each exercise intervention period and the washout period. The children will be asked to maintain normal physical activity patterns and habits throughout the study (supplementary S6). A family caregiver may be present during all procedures, depending on the child's preference, to increase comfort.

### 2.8 Data collection and management

Both quantitative and qualitative data will be collected from all included participants. Feedback from the children and their family caregivers will be collected via the exer-app, via a questionnaire on usability, and through semi-structured interviews (23).

Qualitative data will be collected through questionnaire and semi-structured interviews performed after each exercise period, including the effectiveness testing before and after. The semi-structured interviews will take place at the children's home or through Teams online. The child will be asked to be interviewed first, followed by the caregiver within the same session. The total duration of each session is estimated to be approximately one hour. The supervising personal trainer will be instructed to take fieldnotes after each exercise session and will be semi-structurally interviewed in person (23) after each exercise period. All semi-structured interviews will be audio recorded and field notes documented after by interviewer.

The semi-structured interviews will be performed by a doctoral student under supervision from the research team (TJ: doctoral student, physiotherapist; KL: supervisor, physiotherapist specialized in paediatrics; BAJ: engineer with PhD in computer science; ÅA: professor of biomedical science; OH: geneticist; RH: supervisor, organizational psychologist; ÅBT: main supervisor, physiotherapist specialized in exercise and physical activity). The research team consists of four females and four men in total. Transcripts will not be returned to participants for comments.

Quantitative data will be collected in the exer-app after each exercise session. The children will be asked to fill in information about the perceived exertion of the exercise session, according to the Borg scale (45), and pain, according to the Faces Pain Scale-Revised (46). The number of exercise sessions will be registered through the children marking the session as done in the app. During each exercise session, heart rate and workload will be continuously collected from the bike into the exer-app. Questions about adverse events will be sent out prior to each exercise testing (supplementary S7).

All data collected will be pseudoanonymised and securely stored at Lund University, with access restricted to the research team. Confidentiality will be maintained, and findings reported without traceable individual data.

### 2.9 Data analysis and statistics

A convergent mixed methods approach will be used to assess the feasibility of the exercise intervention, the eHealth solution ability to deliver the exercise interventions and the different procedures in the effectiveness testing from multiple perspectives, integrating both quantitative and qualitative data. In this design, quantitative and qualitative data will be analysed separately and then integrated during interpretation (30).

#### 2.9.1 Feasibility analysis

To structure the feasibility analysis process, scientific, management, and resource (33) uncertainties will be assessed (Table 1). The analysis will have an intent-to-treat (ITT) approach where above 66% of successful cases will be considered feasible, adjustable if successful cases fall between 20% and 65%, and unfeasible if the successful case rate is below 20%, both for individual outcomes and for an overall analysis. Wide thresholds were chosen to allow for iterative refinement of the intervention based on participatory feedback during study. When items are considered adjustable, the data collected from the android tablet computer, field notes, and interviews will be used to further develop the HOME-EX protocol.

**Table 1:** Description of predefined progression criteria.

Items	Outcomes	Predefined progression criteria
***Process:*** This assesses the feasibility of the processes that are key to the success of the main study. Evaluation of study design (e.g., reducing uncertainty around recruitment, data collection, retention, outcomes, and analysis)		
Recruitment	Recruitment rate	>66% of invited participants accepted to participate in the study
		65%–20% of invited participants agreed to participate in the study
		<19% of invited participants agreed to participate in the study
Retention	Retention rate	>66% completed all exercise periods
		65–20% completed all exercise periods
		<19% completed all exercise periods
Data collection	Completion rate and perceived usefulness	>66% completed all procedures and are perceived as useful/acceptable
		65%–20% completed all procedures and perceived them as useful/acceptable
		<19% completed all procedures and perceived them as useful/acceptable
Outcome measures	Variance and distribution of collected data	Outcomes provide interpretable data with acceptable variance
		Outcomes partially interpretable, large variance
		Outcomes are not interpretable, excessive variance
***Scientific:*** This deals with the assessment of treatment safety, dose, response, effect, and variance of the effect (study design)		
Outcome measures	Variance and distribution of collected data	Outcomes provide interpretable data with acceptable variance
		Outcomes partially interpretable, large variance
		Outcomes not interpretable, excessive variance
Acceptability	Perceived acceptability of the eHealth tablet app and home-based exercise	>66% report app/exercise acceptable
		65%–20% report app/exercise acceptable
		<19% report app/exercise acceptable
Usability/technical demands	Technical support needed	Only initial introduction needed
		Required equal time for support as for tablet app use
		Unable to use eHealth app due to technical demands
Barriers/facilitators	Perceived barriers and facilitators when exercising and using eHealth tablet app	Perceived more facilitators than barriers
		Equal numbers of facilitators as barriers
		Perceived more barriers than facilitators
***Management:*** This covers potential human and data management problems		
Capacity to deliver exercise sessions	Trainers' capacity to deliver exercise intervention	>66% completed exercise sessions
		65%–20% completed exercise sessions
		<19% completed exercise sessions
Capacity to deliver exercise equipment	Logistics in delivering cycle ergometers to children's homes	>66% successful deliveries
		65%–20% successful deliveries
		<19% successful deliveries
***Recourses:*** This pertains to assessing time and resource problems that can occur during the main study		
Resources to deliver	Exercise intervention	Trainer reports delivery of exercise sessions as feasible with given resources
		Trainer reports delivery possible but challenging
		Provider reports delivery not feasible
Resources to deliver	Effectiveness testing	The coordination of all tests is feasible within resources
		The coordination of all tests is possible but challenging
		Provider reports delivery not feasible
Resources to deliver	Logistics to deliver cycle ergometers to children's homes	The coordination of all tests is feasible within resources
		The coordination of all tests is possible but challenging
		Provider reports delivery not feasible

Process uncertainties will include as recruitment rate, retention rate, acceptability, usability, barriers/facilitators, and adherence. Scientific uncertainties will include safety, dose, and variance of effect, all aspects related to the study design planned for a future efficacy study, Management uncertainties will address data collection processes, while resource-related uncertainties will assess the time and resource problems involved in running the study (33) (see Table 1 on progress criteria).

#### 2.9.2 Quantitative data

Given the sample size and aim of the feasibility study, quantitative data will be reported descriptively as medians, inter-quartile range, and range. Quantitative data will primarily be used to evaluate feasibility outcomes related to process, including recruitment rate, retention rate, completion of data collection procedures, completion of the exercise intervention, and the variance and distribution of outcome measures (Table 1).

#### 2.9.3 Qualitative data

Qualitative data from field notes and semi-structured interviews will be analysed using thematic content analysis to identify patters and themes related to feasibility outcomes (24). Thematic content analyses refer to the process of recovering themes embodied and dramatized in the evolving meanings and imagery of the transcripts. Two to three coders will be involved in the process. Analysis and interpretation are considered complete when synthesis of the putative experiences seems consistent with the emerging patterns and constitutes a coherent unity.

Qualitative data will primarily be used to explore feasibility outcomes related to acceptability of the intervention, the eHealth solution and the different procedures in the effectiveness testing, usability and technical demands, perceived barriers and facilitators, and experiences related to recruitment, retention, data collection procedures, and the delivery of the exercise intervention (Table 1).

#### 2.9.4 Integration of data

Quantitative and qualitative findings will be integrated during the interpretation phase using triangulation, whereby findings from the qualitative and quantitative data will be compared to identify areas of convergence, complementarity or discrepancy (29, 30). The feasibility analysis will be ongoing throughout the study. After each exercise period the research team will meet and discuss the finding, including participatory feedback from the app and interviews. Depending on the findings gathered during the feasibility analysis, the upcoming exercise periods and effectiveness testing may be changed (Figure 1).

### 2.10 Ethical approval and trail registration

Ethical approval for this research has been received from the Swedish Ethical Review Authority (SERA) Dnr: and 2025-08170-02. Important protocol modifications will be communicated to SERA, ClinicalTrails.gov at (NCT07025694; recruitment in progress) and to relevant parties. All parents and children included in the study will receive a request for informed consent to participate, and for publication. Children aged 15 and above can decide themselves whether or not to participate in the study. Participating child will be insured during the study period.

### 2.11 Dissemination

Results will be reported through peer-reviewed papers, conferences, and popular science articles, with summaries provided to participants.

## 3 Discussion

This study protocol outlines the study's design and methods, including the conceptual framework, data collection procedures, analysis plan, and recruitment strategies. The findings from the study have the potential to contribute to the development and feasibility of home-based exercise interventions supported by eHealth to promote exercise in children with CP-A. In addition, findings from this study will increase our knowledge of the feasibility of a home-based cycling program for young people with CP. The findings will also inform the development of a feasible exercise protocol for future effectiveness studies.

A convergent mixed methods approach was chosen to assess the feasibility of the HOME-EX study from multiple perspectives, using both qualitative and quantitative data. The convergent design allows triangulation, which enhances credibility and interpretability of the results, as areas of convergence or discrepancy can highlight important strengths or limitations in our feasibility study (30). Typically, the study design may risk unequal weighting between qualitative and quantitative outcomes, as quantitative studies need more participants to gain statistical power, potentially decreasing the validity of a mixed methods study (30). In this feasibility study, all study participants will contribute to both the quantitative and qualitative data, mitigating potential unequal weighting of different outcomes. Effectiveness is not studied in the current study, and statistical power is not required for a feasibility study.

In terms of the adapted participatory design, the design is used to continuously account for and include the participants' and their families' needs and everyday context within the feasibility study, strengthening the study design. Building on the participatory design described by Clemensen et al. (31), the first three exercise periods within this feasibility study function as refinement cycles in the second stage (Figure 1), where participatory feedback from the app and interviews will be used to refine the upcoming exercise periods and effectiveness testing. This enables continuous development and testing of the exercise intervention within a single study, while simultaneously enhancing the likelihood that the final version of the exercise intervention, the eHealth solution, and effectiveness testing is well-adapted to children and families before being tested in the fourth exercise period. As the exercise intervention will be under development within the feasibility study, detailed reporting following the Consensus on Exercise Reporting Template (47) including its Explanation and Elaboration Statement (48) will be provided in the manuscript presenting the results of this study.

According to the MRC methodological framework (32), feasibility testing is important, as it makes it possible to assess uncertainties; e.g., the predefined progression criteria, before potentially conducting a larger-scale effectiveness study (32). Within this feasibility study, several predefined progression criteria are set, in terms of quantitative outcomes. Recruitment and retention are well-known challenges in research, which is why progression criteria addressing these aspects are important. As the intervention is delivered in the participants home rather than in a fully equipped training facility, additional criteria addressing delivering, capacity, logistic, and resource demands are essential. Together, these criteria, in line with those described by Thanbane et al. (33), enable a focused assessment of feasibility prior to the progression to a potential future full-scale study. Qualitative outcomes collected from participants will be used when uncertainties are considered adjustable. Information from both quantitative and qualitative outcomes will be used to fine-tune the feasibility of the eHealth solution. When all outcomes are collected, a summary assessment will be conducted to determine the feasibility of the eHealth solutions to deliver an exercise intervention at the children's homes. Although one may argue there are many outcomes, all the outcomes together combine to enhance the understanding and determination of whether the intervention is feasible, thereby reducing the risk of conducting a large multi-centre effectiveness study that was not possible to perform.

The NASSS-CAT framework used for assessing the eHealth solution and the general conditions for implementing the intervention provide a nuanced and robust basis for the identification of risks and opportunities related to the project (34). In addition, the use of the NASSS-CAT framework (34) together with the MRC methodological framework (32) and the convergent mixed methods approach (30) enables the intervention to be examined from multiple perspectives. Altogether, these frameworks (32, 34) and methodological approach (30) support a comprehensive assessment of the complexity of the home-based exercise intervention.

The present feasibility study has limitations that should be considered when interpreting the findings. The small sample size, although appropriate for feasibility testing (36), limits the ability to capture population variability and reduced generalisability. Recruitment of children who are TD through social media platforms may introduce selection bias and potentially limit participation to families with higher literacy or access to online networks (49). The comprehensive testing procedures, including blood samples, may increase participation burden and may influence adherence and retention leading to attrition bias (50). Additionally, in the present study HIIT and MICT periods are not randomized. The absence of randomisation and the sequential design may introduce order and carryover effects in subsequent studies, reducing their internal validity. Finally, wide threshold may overestimate feasibility (51), while participatory design (31) may introduce variability in intervention delivery, reducing standardisation and reproducibility.

Taken together, these limitations highlight potential biases and methodological challenges that should inform the design of subsequent studies. The present feasibility study represents an early step in the research process. A subsequent pilot study is planned if this feasibility study holds promising results. The pilot study will allow for more rigorous testing of procedures and preliminary effectiveness testing. A more stringent feasibility threshold is likely to be applied in the pilot study.

If the present feasibility study and the pilot study show promising results, a larger study evaluating the physiological effectiveness and the cost-effectiveness of HIIT and MICT should consider recruitment from a larger area. This further emphasises the importance of a comprehensive feasibility study.

## 4 Conclusion

By the developing, feasibility testing, and mapping the conditions for future implementation of an eHealth solution to increase exercise for children with CP-A, this study may contribute to future eHealth interventions and support implementation strategies for exercise in children with CP-A, consequently improving their overall health and well-being.

## Ethics statement

The studies involving humans were approved by The Swedish Ethical Review Authority Dnr: and 2025-08170-02. The study will be conducted in accordance with the local legislation and institutional requirements. The participants provided their written informed consent to participate in this study.
