# Supplementary material for: Feasibility testing of a home-based exercise intervention in children with cerebral palsy who are ambulant—a study protocol of the HOME-EX study
Source: Front Digit Health. 2026 May 12;8:1811789. doi: 10.3389/fdgth.2026.1811789 (PMC13202785; doi:10.3389/fdgth.2026.1811789)
Supplement: Supplementary file 1 [file Datasheet1.docx]

Supplementary Material

**Supplement 1**

**SPIRIT 2025 checklist of items to address in a randomized trial protocol***

| **Section / Topic** | **No** | **SPIRIT 2025 checklist item description** | **Reported on page no.** |
| --- | --- | --- | --- |
| **Administrative information** | | |  |
| Title and structured summary | 1a | Title stating the trial design, population, and interventions, with identification as a protocol | Title page |
|  | 1b | Structured summary of trial design and methods, including items from the World Health Organization Trial Registration Data Set | Abstract |
| Protocol version | 2 | Version date and identifier | Abstract |
| Roles and responsibilities | 3a | Names, affiliations, and roles of protocol contributors | Title page,  6 Author Contributions |
|  | 3b | Name and contact information for the trial sponsor | N/A |
|  | 3c | Role of trial sponsor and funders in design, conduct, analysis, and reporting of trial; including any authority over these activities | 7 Funding |
|  | 3d | Composition, roles, and responsibilities of the coordinating site, steering committee, endpoint adjudication committee, data management team, and other individuals or groups overseeing the trial, if applicable | 2.8 Data collection and Management.  2.9.3 Qualitative Data.  2.9.4 Integration of Data. |
| **Open science** | | |  |
| Trial registration | 4 | Name of trial registry, identifying number (with URL), and date of registration. If not yet registered, name of intended registry | 2.10 Ethical Approval and Trail Registration |
| Protocol and statistical analysis plan | 5 | Where the trial protocol and statistical analysis plan can be accessed | 2.10 Ethical Approval and Trail Registration |
| Data sharing | 6 | Where and how the individual de-identified participant data (including data dictionary), statistical code, and any other materials will be accessible | 9 Data Availability Statement |
| Funding and conflicts of interest | 7a | Sources of funding and other support (e.g., supply of drugs) | 7 Funding |
|  | 7b | Financial and other conflicts of interest for principal investigators and steering committee members | 5 Conflict of Interest |
| Dissemination policy | 8 | Plans to communicate trial results to participants, healthcare professionals, the public, and other relevant groups (e.g., reporting in trial registry, plain language summary, publication) | 2.11 Dissemination |
| **Introduction** | | |  |
| Background and rationale | 9a | Scientific background and rationale, including summary of relevant studies (published and unpublished) examining benefits and harms for each intervention | 1 Introduction |
|  | 9b | Explanation for choice of comparator | N/A |
| Objectives | 10 | Specific objectives related to benefits and harms | Table 1. |
| **Methods: Patient and public involvement, trial design** | | |  |
| Patient and public involvement | 11 | Details of, or plans for, patient or public involvement in the design, conduct, and reporting of the trial | 2.1 Design,  2.9.4 Integration of Data,  Figure 1. |
| Trial design | 12 | Description of trial design including type of trial (e.g., parallel group, crossover), allocation ratio, and framework (e.g., superiority, equivalence, non-inferiority, exploratory) | 2.5 Home-based Exercise Intervention |
| **Methods: Participants, interventions, and outcomes** | | |  |
| Trial setting | 13 | Settings (e.g., community, hospital) and locations (e.g., countries, sites) where the trial will be conducted | 2.4 Settings |
| Eligibility criteria | 14a | Eligibility criteria for participants | 2.3 Participants |
|  | 14b | If applicable, eligibility criteria for sites and for individuals who will deliver the interventions (e.g., surgeons, physiotherapists) | N/A |
| Intervention and comparator | 15a | Intervention and comparator with sufficient details to allow replication including how, when, and by whom they will be administered. If relevant, where additional materials describing the intervention and comparator (e.g., intervention manual) can be accessed | 2.5 Home-based Exercise Intervention  2.6 eHealth Solution  2.7 Feasibility of the Effectiveness Testing of the Exercise Intervention |
|  | 15b | Criteria for discontinuing or modifying allocated intervention/comparator for a trial participant (e.g., drug dose change in response to harms, participant request, or improving/worsening disease) | N/A |
|  | 15c | Strategies to improve adherence to intervention/comparator protocols, if applicable, and any procedures for monitoring adherence (e.g., drug tablet return, sessions attended) | 2.6 eHealth Solution  2.7 Feasibility of the Effectiveness Testing of the Exercise Intervention |
|  | 15d | Concomitant care that is permitted or prohibited during the trial | N/A |
| Outcomes | 16 | Primary and secondary outcomes, including the specific measurement variable (e.g., systolic blood pressure), analysis metric (e.g., change from baseline, final value, time to event), method of aggregation (e.g., median, proportion), and time point for each outcome | 2.9.1 Feasibility Analysis  Table 1. |
| Harms | 17 | How harms are defined and will be assessed (e.g., systematically, non-systematically) | 2.8 Data collection and Management, Table 1. |
| Participant timeline | 18 | Time schedule of enrollment, interventions (including any run-ins and washouts), assessments, and visits for participants. A schematic diagram is highly recommended (see Figure) | Figure 3. |
| Sample size | 19 | How sample size was determined, including all assumptions supporting the sample size calculation | 2.3 Participants |
| Recruitment | 20 | Strategies for achieving adequate participant enrollment to reach target sample size | 2.3 Participants |
| **Methods: Assignment of interventions** | | |  |
| Randomization: |  |  |  |
| Sequence generation | 21a | Who will generate the random allocation sequence and the method used | N/A |
|  | 21b | Type of randomization (simple or restricted) and details of any factors for stratification. To reduce predictability of a random sequence, other details of any planned restriction (e.g., blocking) should be provided in a separate document that is unavailable to those who enroll participants or assign interventions | N/A |
| Allocation concealment  mechanism | 22 | Mechanism used to implement the random allocation sequence (e.g., central computer/telephone; sequentially numbered, opaque, sealed containers), describing any steps to conceal the sequence until interventions are assigned | N/A |
| Implementation | 23 | Whether the personnel who will enroll and those who will assign participants to the interventions will have access to the random allocation sequence | N/A |
| Blinding | 24a | Who will be blinded after assignment to interventions (e.g., participants, care providers, outcome assessors, data analysts) | N/A |
|  | 24b | If blinded, how blinding will be achieved and description of the similarity of interventions | N/A |
|  | 24c | If blinded, circumstances under which unblinding is permissible, and procedure for revealing a participant’s allocated intervention during the trial | N/A |
| **Methods: Data collection, management, and analysis** | | |  |
| Data collection methods | 25a | Plans for assessment and collection of trial data, including any related processes to promote data quality (e.g., duplicate measurements, training of assessors) and a description of trial instruments (e.g., questionnaires, laboratory tests) along with their reliability and validity, if known. Reference to where data collection forms can be accessed, if not in the protocol | 2.8 Data collection and Management. |
|  | 25b | Plans to promote participant retention and complete follow-up, including list of any outcome data to be collected for participants who discontinue or deviate from intervention protocols | 2.6 eHealth Solution  Table 1. |
| Data management | 26 | Plans for data entry, coding, security, and storage, including any related processes to promote data quality (e.g., double data entry; range checks for data values). Reference to where details of data management procedures can be accessed, if not in the protocol | 2.8 Data collection and Management. |
| Statistical methods | 27a | Statistical methods used to compare groups for primary and secondary outcomes, including harms | 2.9.2 Quantitative Data |
|  | 27b | Definition of who will be included in each analysis (e.g., all randomized participants), and in which group | 2.9.2 Quantitative Data |
|  | 27c | How missing data will be handled in the analysis | 2.9.2 Quantitative Data |
|  | 27d | Methods for any additional analyses (e.g., subgroup and sensitivity analyses) | N/A |
| **Methods: Monitoring** | | |  |
| Data monitoring committee | 28a | Composition of data monitoring committee (DMC); summary of its role and reporting structure; statement of whether it is independent from the sponsor and funder; conflicts of interest and reference to where further details about its charter can be found, if not in the protocol. Alternatively, an explanation of why a DMC is not needed | N/A |
|  | 28b | Explanation of any interim analyses and stopping guidelines, including who will have access to these interim results and make the final decision to terminate the trial | N/A |
| Trial monitoring | 29 | Frequency and procedures for monitoring trial conduct. If there is no monitoring, give explanation | N/A |
| **Ethics** | | |  |
| Research ethics approval | 30 | Plans for seeking research ethics committee/institutional review board approval | 2.10 Ethical Approval and Trail Registration |
| Protocol amendments | 31 | Plans for communicating important protocol modifications to relevant parties | 2.10 Ethical Approval and Trail Registration |
| Consent or assent | 32a | Who will obtain informed consent or assent from potential trial participants or authorized proxies, and how | 2.10 Ethical Approval and Trail Registration |
|  | 32b | Additional consent provisions for collection and use of participant data and biological specimens in ancillary studies, if applicable | N/A |
| Confidentiality | 33 | How personal information about potential and enrolled participants will be collected, shared, and maintained in order to protect confidentiality before, during, and after the trial | 2.10 Ethical Approval and Trail Registration  9 Data Availability Statement |
| Ancillary and post-trial care | 34 | Provisions, if any, for ancillary and post-trial care, and for compensation to those who suffer harm from trial participation | 2.10 Ethical Approval and Trail Registration |

*We strongly recommend reading this checklist in conjunction with the SPIRIT 2025 Explanation and Elaboration and the SPIRIT 2025 Expanded Checklist for important clarifications on all the items. We also recommend reading relevant SPIRIT extensions. See [www.consort-spirit.org](http://www.consort-spirit.org)

Citation: Chan A-W, Boutron I, Hopewell S, Moher D, Schulz KF, et al. SPIRIT 2025 statement: updated guideline for protocols of randomised trials. BMJ 2025;389:e081477. <https://dx.doi.org/10.1136/bmj-2024-081477>

© 2025 Chan A-W et al. This is an Open Access article distributed under the terms of the Creative Commons Attribution License (<https://creativecommons.org/licenses/by/4.0/>), which permits unrestricted use, distribution, and reproduction in any medium, provided the original work is properly cited.

**Supplement 2.**

| **Consolidated criteria for reporting qualitative studies (COREQ): 32-item checklist** | | | |
| --- | --- | --- | --- |
|  | **Item** | **Guide questions/description** | **Page** |
| **Domain 1: Research team and reflexivity** |  |  |  |
| Personal Characteristics |  |  |  |
| 1. | Interviewer/facilitator | Which author/s conducted the interview or focus group? | 2.8 Data collection and Management |
| 2. | Credentials | What were the researcher's credentials? *E.g. PhD, MD* | 2.8 Data collection and Management |
| 3. | Occupation | What was their occupation at the time of the study? | 2.8 Data collection and Management |
| 4. | Gender | Was the researcher male or female? | 2.8 Data collection and Management |
| 5. | Experience and training | What experience or training did the researcher have? | 2.8 Data collection and Management |
| Relationship with participants |  |  |  |
| 6. | Relationship established | Was a relationship established prior to study commencement? | 2.8 Data collection and Management |
| 7. | Participant knowledge of the interviewer | What did the participants know about the researcher? e*.g. personal goals, reasons for doing the research* | 2.8 Data collection and Management |
| 8. | Interviewer characteristics | What characteristics were reported about the interviewer/facilitator? e.g. *Bias, assumptions, reasons and interests in the research topic* | 2.8 Data collection and Management |
| **Domain 2: study design** |  |  |  |
| Theoretical framework |  |  |  |
| 9. | Methodological orientation and Theory | What methodological orientation was stated to underpin the study? *e.g. grounded theory, discourse analysis, ethnography, phenomenology, content analysis* | 2.9.3 Qualitative Data |
| Participant selection |  |  |  |
| 10. | Sampling | How were participants selected? *e.g. purposive, convenience, consecutive, snowball* | 2.3 Participants |
| 11. | Method of approach | How were participants approached? e*.g. face-to-face, telephone, mail, email* | 2.3 Participants |
| 12. | Sample size | How many participants were in the study? | 2.3 Participants  2.8 Data collection and Management |
| 13. | Non-participation | How many people refused to participate or dropped out? Reasons? | N/A |
| Setting |  |  |  |
| 14. | Setting of data collection | Where was the data collected? e*.g. home, clinic, workplace* | 2.8 Data collection and Management |
| 15. | Presence of non-participants | Was anyone else present besides the participants and researchers? | N/A |
| 16. | Description of sample | What are the important characteristics of the sample? *e.g. demographic data, date* | 2.3 Participants 2.4 Settings |
| Data collection |  |  |  |
| 17. | Interview guide | Were questions, prompts, guides provided by the authors? Was it pilot tested? | N/A |
| 18. | Repeat interviews | Were repeat interviews carried out? If yes, how many? | 2.8 Data collection and Management |
| 19. | Audio/visual recording | Did the research use audio or visual recording to collect the data? | 2.8 Data collection and Management |
| 20. | Field notes | Were field notes made during and/or after the interview or focus group? | 2.8 Data collection and Management |
| 21. | Duration | What was the duration of the interviews or focus group? | 2.8 Data collection and Management |
| 22. | Data saturation | Was data saturation discussed? | 2.3 Participants |
| 23. | Transcripts returned | Were transcripts returned to participants for comment and/or correction? | 2.8 Data collection and Management |
| **Domain 3: analysis and findings** |  |  |  |
| Data analysis |  |  |  |
| 24. | Number of data coders | How many data coders coded the data? | 2.9.3 Qualitative Data |
| 25. | Description of the coding tree | Did authors provide a description of the coding tree? | N/A |
| 26. | Derivation of themes | Were themes identified in advance or derived from the data? | N/A |
| 27. | Software | What software, if applicable, was used to manage the data? | N/A |
| 28. | Participant checking | Did participants provide feedback on the findings? | 2.9.3 Qualitative Data |
| Reporting |  |  |  |
| 29. | Quotations presented | Were participant quotations presented to illustrate the themes / findings? Was each quotation identified? e*.g. participant number* | N/A |
| 30. | Data and findings consistent | Was there consistency between the data presented and the findings? | N/A |
| 31. | Clarity of major themes | Were major themes clearly presented in the findings? | N/A |
| 32. | Clarity of minor themes | Is there a description of diverse cases or discussion of minor themes? | N/A |

**Supplement 3**

| **Good Reporting of A Mixed Methods Study (GRAMMS)** | |
| --- | --- |
|  | Pages |
| 1. Describe the justification for using a mixed methods approach to the research question | 2.9 Data Analysis and Statistics |
| 1. Describe the design in terms of the purpose, priority and sequence of methods | 2.1 Design  2.8 Data collection and Management  2.9 Data Analysis and Statistics |
| 1. Describe each method in terms of sampling, data collection and analysis | 2.3 Participants  2.8 Data collection and Management  2.9 Data Analysis and Statistics |
| 1. Describe where integration has occurred, how it has occurred and who has participated in it | 2.9.4 Integration of Data |
| 1. Describe any limitation of one method associated with the present of the other method | 3 Discussion |
| 1. Describe any insights gained from mixing or integrating methods | N/A |

# Supplement 4

pROM was assessed for abduction, flexion, extension, Ely’s test, internal rotation, and

external rotation in the hip according to the CPUP protocol (www.cpup.se), using a handheld goniometer.

Spasticity in hip flexors, extensors and adductors, was assessed by the Modified Ashworth Scale. This scale is as follows:

0 No increase in muscle tone

1 Slight increase in muscle tone, manifests itself as "catch and release", or as minimum resistance at the end of the motion path

+1 Slight increase in muscle tone, appears as a "catch", followed by minimal resistance through the rest of the motion path (less than half the range of motion)

2 More marked increase of muscle tone through most of the motion path, but the movement is still easy to perform

3 Significant increase in muscle tone, difficult to perform passive movements

4 Stiffness when trying to flex or extend a body part.

Starting positions used when estimating spasticity when lying in a supine position.

Starting positions used when estimating spasticity when lying in a supine position.

*Hip flexors:* the leg was moved in a flexion–extension range of motion to feel the muscle tone when the hip was extended.

*Hip extensors:* the leg was moved in a flexion–extension range of motion to feel the muscle tone when the hip was flexed.

*Adductors:* assessed with the child’s hips and knees extended. The leg was moved in an abduction muscle tonus adduction range of motion, to feel the muscle tone when the leg was abducted.

# Supplement 5

*Biomarkers for axis* *of exercise-induced neurologic, endocrine, and immunological changes measured in blood*

Venous and capillary blood samples will be collected before and after the exercise tests. Before coming to the lab, the children will use EMLA plaster (Aspen Pharma Trading Limited, Dublin, Ireland) containing 25mg lidocaine and 25mg prilocaine as pain relief, on the back of their hands and on their arm folds. The EMLA plaster should be applied at each of the four sites at least one hour, and no more than five hours, before the lab visit.

Biomarkers will be measured using proteomics (Olink Explore). Changes in biomarkers will be assessed in the following categories: 1) neural growth factors, e.g., BDNF, VEGF and NGF; 2) the IGF-axis, e.g., GH, IGF-1 and IGFBP-3; 3) the HPA-axis, e.g., ACTH, cortisol, adrenaline, and noradrenaline; 4) inflammatory markers, e.g., CRP, TNF-α and IL-6; and 5) insulin sensitivity for blood glucose and for blood-free fatty acid metabolism, e.g., insulin, glucose, lactate, and free fatty acids. Biomarkers, which are up- or downregulated in response to exercise, will be identified by comparing changes in biomarker concentrations in response to HIIT/MICT between ambulant children with CP-A and TD children.

All blood samples will be stored at –80 °C and analysed by Olink or ELISA at the same time to avoid any effects from different reagent batches. Olink results will also be used for an exploratory analysis of additional biomarkers for individualisation of the exercise training approach.

# Supplement 6

*Exercise testing*

Indirect calorimetry will be used during a single session of high intensity exercise; i.e., during a combined steady state and progressive incremental exercise test until exhaustion (Figure 3), to assess markers of ventilation, circulation, fat versus carbohydrate oxidation, and energy expenditure during exercise.

Exercise tests will be performed on a cycle ergometer (LC6, Monark Exercise AB, Sweden). An airtight mask covering the mouth and nose will be worn to measure breath-by-breath VO_2_ and VCO_2_, for determination of VO_2_peak and Respiratory Exchange Ratio (RER) (Vyntus CXP, Jaeger, Germany). Heart rate will be recorded continuously throughout the test (Polar T1, Polar, Finland). For familiarisation purposes, all children will watch a movie wearing the facial mask for five minutes before the exercise testing begins.

The combined steady state and progressive incremental exercise test until exhaustion is described in Figure 3. This exercise test will be initiated by cycling on a cycle ergometer for six minutes at 30W, followed by an increase of 10W/minute until exhaustion (see Figure 3).

# Supplement 7

Information collected in android computer tablet.

| **Planning**: all calendar entries for patients. | We extract the following data for each patient and calendar entry:   - Patient ID (serial number) - Start time (date, time) - End time (date, time) - Type (text) - Event (text) - Description (text) - Can patient mark it as completed? (binary) - Is it completed? (binary) - Latest change (date, time) |
| --- | --- |
| **Communication**: all chat messages to and from patients. | We extract the following data for each patient and chat message to or from the patient:   - Patient ID (serial number) - Staff ID, sender (serial number) *if applicable* - Message time (date, time) - Handling status ('unhandled', 'handling', 'handled', 'revoked') - Staff ID, handler (serial number) *if applicable* |
| **Assessments**: all bike exercise assessments (v1.0) registered by patients. | We extract the following data for each patient and assessment registration:   - Patient ID (serial number) - Registration time (date, time) - Registration comment (text) - Adhered to instruction? (binary) - Comment, adherence (text) - Duration, minutes (integer) - Exertion, Borg scale (integer) - Comment, exertion (text) - Satisfaction (‘great’, ‘good’, ‘bad’) - Pain, Faces Pain Scale-Revised (integer) - Comment, pain (text) - Signature, initials (text) |
| **Exercise data**: all bike and heart rate data registered in the Monark app. | The following data is continuously recorded from the bikes and heart rate monitors in the manufacturer’s (Monark) app for each patient and bike exercise session:   - Session time (seconds) - Unix time (milliseconds) - Heart rate (bpm) - Age, heart rate (nanoseconds) - Power (W) - Resistance (kp) - Cadence (rpm) - Speed (km/h) - Distance (m) - Energy expenditure (kcal) |
